# Are Saturday Fund Subscribers Entitled to Free Medical Relief?

**Published:** 1913-02-15

**Authors:** A. Charles E. Gray

**Affiliations:** 2 Stanhope Gardens, South Kensington, S.W.


### The Editor's Letter-Box.

### Are Saturday Fund Subscribers entitled to Free Medical Relief?

*To the Editor of* The Hospital.

Sir,—Your correspondent, Mr. Lewcock, of the Hospital Saturday Fund, raises a point of very great importance both to hospital committees and the cause of charity in general. He asks the question, "Are our subscribers entitled to treatment in respect to the amount subscribed by them to the hospital?" My answer, and I trust that of every hospital manager, is an emphatic No. If the subscription to the hospital is in the nature of a contract payment for medical attendance, then the hospital is not a charity, and the payment is in most instances ridiculously inadequate; if it is not a contract, then the hospital is not only justified, but in duty bound to make inquiries as to fitness of the recipients of the charity. Fortunately for the patients, however, the hospitals are charities.

If the manner in which inquiries are made leaves something to be desired, the sooner that is remedied the better; but if, as I suspect, it is the inquiries themselves which are resented, then the sooner the working-man subscriber learns that he is assisting a charity and not making a payment for services the better for all concerned.—I am, yours truly, A. Charles E. Gray, M.D.

2 Stanhope Gardens, South Kensington, S.W., February 9, 1913.

